# Impact of pre-therapeutic fasting plasma glucose on survival outcomes in advanced non-small cell lung cancer patients

**DOI:** 10.3389/fendo.2025.1630503

**Published:** 2026-01-09

**Authors:** Feiwen Liu, Min Luo, Fang Wang, Ting Liang, Xiaochen Wang

**Affiliations:** 1 Department of Oncology, The Third Affiliated Hospital of Guangxi Medical University, Nanning, Guangxi Zhuang Autonomous Region, China; 2 Department of Rheumatology and Immunology, Foresea Life Insurance Guangxi Hospital, Nanning, Guangxi Zhuang Autonomous Region, China

**Keywords:** fasting plasma glucose (FPG), glycemic management, non-small cell lung cancer (NSCLC), prognostic biomarker, smooth curve fitting

## Abstract

**Purpose:**

The necessity of tight glycemic management in non-small cell lung cancer (NSCLC) remains controversial. This study aimed to determine whether baseline fasting plasma glucose (FPG) levels could serve as an independent prognostic marker for survival outcomes in advanced NSCLC.

**Patients and methods:**

This study included 960 patients with advanced NSCLC, who were categorized into low (< 3.9 mmol/L), normal (3.9–6.1 mmol/L), and high FPG groups (> 6.1 mmol/L) based on pre-treatment FPG levels. The analyzed covariates included demographics, clinical characteristics, oncogenic mutation status, and first-line treatments. Survival curves with log-rank tests were estimated to compare survival differences between groups. Univariate and multivariate Cox proportional hazards regression were performed to investigate the prognostic factors. Furthermore, smooth curve fitting and piecewise Cox regression were used to explore the non-linear relationships between FPG and mortality risk, while subgroup analyses were employed to test interactions.

**Results:**

Both low (12.0 *vs*. 18.5 months, *P* = 0.0093) and high (14.4 *vs*. 18.5 months, *P* = 0.0049) FPG levels were significantly associated with shorter median survival times compared to normal FPG levels. Multivariable analyses further identified low FPG (HR 1.41, 95% CI 1.06–1.88; *P* = 0.0196) and high FPG (HR 1.43, 95% CI 1.11–1.85; *P* = 0.0059) as independent prognostic risk factors. Smooth curve fitting and piecewise Cox proportional hazards models revealed a negative linear relationship between FPG levels and mortality risk (HR 0.70, 95% CI 0.52–0.94; *P* = 0.0185) when FPG was below the breakpoint of 4.46 mmol/L, and a positive linear relationship (HR 1.11, 95% CI 1.04–1.19; *P* = 0.0015) when FPG exceeded the breakpoint. Subgroup analyses consistently supported these findings across all patient subgroups, with no specific population exhibiting distinct outcomes.

**Conclusion:**

Abnormal FPG levels are independent risk factors for the long-term prognosis of advanced NSCLC. Further prospective multicenter studies are needed to confirm these associations and clarify whether glycemic assessment and management influence survival outcomes.

## Introduction

Lung cancer remains one of the most significant global health challenges. Among its subtypes, non-small cell lung cancer (NSCLC) accounts for approximately 85% of all cases and frequently presents at an advanced stage, where curative treatment options are limited (1). Despite advancements in targeted therapies and immunotherapies, the prognosis of advanced metastatic NSCLC remains poor, with five-year survival rates hovering around 0-10% (2). Identifying prognostic factors that improve patient stratification and guide treatment decisions is an ongoing focus of clinical research. Clinicians currently rely on well-recognized factors, such as patients’ smoking history, histologic classification, TNM staging, Eastern Cooperative Oncology Group performance status (ECOG-PS), and specific genetic changes, such as epidermal growth factor receptor (EGFR) or anaplastic lymphoma kinase (ALK) mutations, to predict outcomes and tailor treatment plans. However, recent research has begun to highlight the potential role of metabolic imbalances in shaping cancer progression, with elevated fasting plasma glucose (FPG) levels emerging as a key area of interest. FPG, a seemingly straightforward metabolic parameter, may have implications for cancer biology and patient outcomes (3–7).

Cancer progression is closely linked to metabolic dysregulation, a hallmark of cancer identified by Warburg nearly a century ago (8). Tumor cells exhibit heightened glucose metabolism to fuel their growth and survival; however, systemic metabolic changes in patients with cancer may also reflect disease burden and progression (9, 10). FPG, a commonly measured clinical parameter, has been recognized for its role in predicting outcomes in certain cancer types, such as pancreatic, bladder, colorectal, breast, and cervical cancer (3–7). These effects may be mediated by various mechanisms, including chronic inflammatory signaling, insulin resistance, and the interplay between glucose availability and tumor metabolism (11). However, findings regarding the prognostic significance of FPG in NSCLC remain sparse.

Several studies have linked hyperglycemia with worse outcomes in patients with cancer, suggesting that elevated glucose levels may accelerate tumor progression. Conversely, emerging evidence also demonstrates that hypoglycemia, reflective of malnutrition or cachexia, may identify patients with particularly aggressive disease or an advanced systemic burden (9, 12). This paradox highlights the complex bidirectional relationship between FPG levels and cancer biology, necessitating further investigation. In advanced NSCLC, most prior studies have focused on driver gene-positive patients (e.g., those with EGFR mutations or ALK rearrangements) (13, 14). Driver gene-negative patients often rely on chemotherapy or chemoimmunotherapy as frontline treatments (15). However, the impact of pretreatment FPG levels on the prognosis of NSCLC patients remains relatively understudied. This lack of focus is particularly notable, given the potential metabolic interactions that may influence cancer progression and treatment outcomes. Thus, the relationship between FPG and outcomes in this understudied cohort warrants further exploration.

This study leveraged real-world data (RWD), a rich source of information drawn from routine clinical practice, to overcome these challenges. Unlike controlled clinical trials that enroll narrowly defined patient groups, RWD reflects the diversity of real-life populations and complex decisions made in everyday oncology care. Our analysis focused on 960 individuals diagnosed with advanced NSCLC, who were divided into three categories based on pre-treatment FPG measurements: low (< 3.9 mmol/L), normal (3.9–6.1 mmol/L), and high (> 6.1 mmol/L). We propose that both abnormally low and high FPG levels serve as independent predictors of survival.

In summary, this study provides new insights into the prognostic value of FPG levels in patients with advanced NSCLC. By addressing gaps in the existing research, we aim to highlight the clinical relevance of metabolic factors in this population and provide a basis for future studies exploring the underlying biological mechanisms. Understanding the complex relationship between systemic metabolism and tumor behavior is critical for improving outcomes in patients with advanced NSCLC.

## Materials and methods

### Study design and population

This retrospective cohort study analyzed patients who were newly diagnosed with advanced NSCLC, defined as surgically unresectable stage III or stage IV disease, at The Third Affiliated Hospital of Guangxi Medical University between January 31, 2010, and January 31, 2020. A total of 1169 patients were initially screened for their eligibility. The exclusion criteria were as follows: (1) absence of pre-treatment FPG records, (2) loss to follow-up at the time of diagnosis, (3) diagnosis of small cell lung cancer, (4) presence of secondary malignancies, (5) abnormally low or high pre-treatment FPG values that may not be properly recorded, and (6) acute metabolic disturbances (e.g., active infections or recent steroid use) likely to transiently affect FPG levels. After applying these criteria, 960 patients were included in the final analysis.

This study received approval from the Institutional Review Board of The Third Affiliated Hospital of Guangxi Medical University (document number: Y2024089), and was conducted in accordance with the ethical principles of the Declaration of Helsinki. As a retrospective analysis utilizing anonymized clinical data, this study posed no interference with treatment protocols or risks to patient welfare. All patient records were de-identified at the source and maintained in password-protected databases with access restricted to study investigators. Given the observational nature of this study and the complete anonymization of the datasets, the ethics committee waived the requirement for informed consent.

### Data collection

The data sources consisted of electronic medical records supplemented by additional information collected through telephone follow-ups. All baseline FPG test samples were uniformly obtained via venous blood draws at 5 a.m. FPG levels were nonrandomly classified into three categories based on standard clinical reference ranges: low FPG (< 3.9 mmol/L), normal FPG (3.9–6.1 mmol/L), and high FPG (> 6.1 mmol/L) (16). Covariates included baseline demographic and clinical characteristics, as well as treatment-related variables, such as sex, age, smoking status (defined as > 100 lifetime cigarettes), ECOG-PS, body mass index (BMI), tumor histology, and clinical staging according to the 8th edition of the International Association for the Study of Lung Cancer TNM classification system (2). Additional variables included metastatic burden (number of metastatic sites), EGFR and ALK mutation status (determined via real-time polymerase chain reaction or next-generation sequencing), and first-line treatment regimens selected in accordance with evidence-based guidelines. To reduce potential bias during the study, the investigators were blinded to the FPG group assignments during data analysis and survival outcome assessments, ensuring objectivity in the interpretation of the results.

Overall survival (OS) data were tracked from treatment initiation until death or the last follow-up (January 31, 2025). Survival duration was calculated from the pathological diagnosis to either death or censoring, with a maximum follow-up period of 5 years. Follow-up intervals were systematically documented using hospital records and direct patient/family communication, maintaining assessment gaps of ≤ 3 months to reduce loss-to-follow-up bias.

### Statistical analysis

Descriptive statistics were used to summarize the baseline characteristics of the study cohort across the three FPG categories. Age was stratified into two groups (> 60 and ≤ 60 years), and BMI was categorized into three groups (< 18.5, 18.5–24.9, and ≥ 25) based on clinically relevant thresholds. Metastatic sites were dichotomized as ≤ 2 versus > 2 using established prognostic cutoffs (17). Comparisons across the three FPG groups were conducted using appropriate Chi-square or Fisher’s exact tests for categorical variables. The associations between potential risk factors and OS were assessed using univariate Cox proportional hazards regression analysis. Variables with *P* < 0.05 in the univariate analysis were included in the multivariate Cox proportional hazards model to estimate the adjusted hazard ratios (HRs) and 95% confidence intervals (CIs). In this study, generalized additive models (GAM) were applied to assess the non-linear associations between the outcome variable and potential risk factors. Curve fitting was conducted using restricted cubic splines (RCS), defined via the rcs function in the rms package in R. The Cox proportional hazards model was then used to generate fitted curves for the log (HR) values. A piecewise Cox regression model was employed to explore potential threshold or saturation effects. The log-likelihood ratio test was used to compare the linear and nonlinear models to identify the best-fitting approach. Kaplan-Meier survival curves, accompanied by log-rank tests, were generated to visualize survival differences among the three FPG groups. Additionally, subgroup-stratified forest plots and interaction tests were used to assess the consistency of the association between FPG and overall survival across various clinical subsets. All statistical analyses were performed using R software (version 3.4.3; http://www.R-project.org) combined with EmpowerStats (version 2023; http://www.empowerstats.com). A two-sided *p*-value threshold of < 0.05 was established to determine statistical significance throughout the study.

## Results

### Selected patients and baseline characteristics

A total of 960 patients with advanced NSCLC were included in this retrospective cohort study (**Supplementary Figure S1**). Based on FPG levels, patients were categorized into three groups: low FPG (n = 94), normal FPG (n = 757), and high FPG (n = 109). The patients’ ages ranged from 25.0 to 88.3 years, with a mean age of 57.9 ± 10.7. The overall median follow-up time was 20.7 months, and the median OS time for the cohort was 17.2 months. Most patients were classified as having adenocarcinoma or squamous cell carcinoma, while 36 patients (3.8%) had other pathological subtypes, including 24 cases of adenosquamous carcinoma, 8 cases of large cell carcinoma, and 4 cases of unspecified carcinoma.

The detailed clinical and pathological characteristics of the study cohort stratified by FPG levels are summarized in **Table 1**. High FPG levels were significantly associated with older age (*P* = 0.009) and elevated BMI (*P* = 0.041), whereas low FPG levels were significantly associated with smoking (*P* = 0.020). In contrast, no significant differences were observed among the FPG groups for other baseline characteristics, including sex, ECOG-PS, histological subtype, clinical stage, number of metastatic sites, EGFR/ALK mutation status, and first-line treatment regimens. This cohort represents a diverse and well-characterized patient population reflective of real-world clinical settings, providing a robust foundation for analyzing the impact of FPG levels on overall survival in advanced NSCLC.

**Table 1:** Clinicopathological characteristics of enrolled patients stratified by FPG.

Patient Characteristics	Low FPG (n=94)	Normal FPG (n=757)	High FPG (n=109)	*P*-value
Sex				0.321
Female	27 (28.72%)	269 (35.54%)	34 (31.19%)	
Male	67 (71.28%)	488 (64.46%)	75 (68.81%)	
Age (years)				**0.009**
≤ 60	50 (53.19%)	436 (57.60%)	46 (42.20%)	
> 60	44 (46.81%)	321 (42.40%)	63 (57.80%)	
Smoking status				**0.020**
No	36 (38.30%)	402 (53.10%)	60 (55.05%)	
Yes	58 (61.70%)	355 (46.90%)	49 (44.95%)	
ECOG-PS				0.709
0-1	88 (93.62%)	690 (91.15%)	99 (90.83%)	
2-3	6 (6.38%)	67 (8.85%)	10 (9.17%)	
BMI (kg/m²)				**0.041**
< 18.5	21 (22.34%)	109 (14.40%)	8 (7.34%)	
≥ 25	11 (11.70%)	102 (13.47%)	19 (17.43%)	
18.5-24.9	62 (65.96%)	546 (72.13%)	82 (75.23%)	
Histological type				0.869
Adenocarcinomas	67 (71.28%)	555 (73.32%)	84 (77.06%)	
Squamous carcinoma	24 (25.53%)	173 (22.85%)	21 (19.27%)	
Others	3 (3.19%)	29 (3.83%)	4 (3.67%)	
Stage				0.272
III	16 (17.02%)	119 (15.72%)	11 (10.09%)	
IV	78 (82.98%)	638 (84.28%)	98 (89.91%)	
Number of metastatic sites				0.608
≤ 2	67 (71.28%)	574 (75.83%)	76 (69.72%)	
> 2	20 (21.28%)	129 (17.04%)	23 (21.10%)	
Unknown	7 (7.45%)	54 (7.13%)	10 (9.17%)	
EGFR/ALK mutation				0.841
No	23 (24.47%)	149 (19.68%)	21 (19.27%)	
Yes	12 (12.77%)	112 (14.80%)	15 (13.76%)	
Unknown	59 (62.77%)	496 (65.52%)	73 (66.97%)	
First-line treatment				0.588
Chemotherapy	40 (42.55%)	384 (50.73%)	56 (51.38%)	
TKIs	20 (21.28%)	136 (17.97%)	25 (22.94%)	
Combination therapy	9 (9.57%)	57 (7.53%)	7 (6.42%)	
Unknown	25 (26.60%)	180 (23.78%)	21 (19.27%)	

*P* values were calculated through the chi-square test or Fisher’s exact test. *P* values <0.05 were highlighted in bold. FPG, Fasting Plasma Glucose; ECOG PS, Eastern Cooperative Oncology Group Performance Status; BMI, body mass index; EGFR, epidermal growth factor receptor; ALK, Anaplastic Lymphoma Kinase; TKIs, tyrosine kinase inhibitors. Combination therapy in this study was defined as chemotherapy combined with anti-angiogenic therapy or immunotherapy.

### Effect of potential predictors on OS

**Figure 1:**
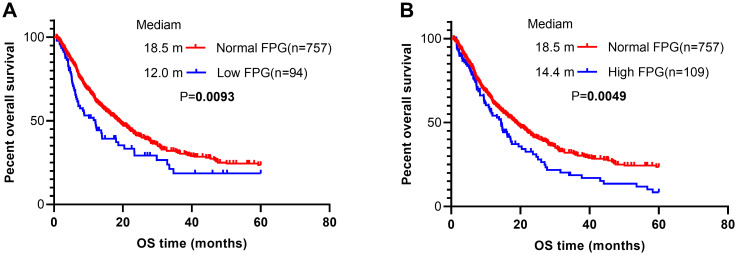
Kaplan–Meier (KM) curves according to different pretreated FPG levels. **(A)** overall survival curves of all patients stratified by low and normal FPG levels; **(B)** overall survival curves of all patients stratified by high and normal FPG levels.

In the univariate analysis (**Table 2**), several factors were significantly associated with an increased risk of mortality. These included male sex (HR 1.31, 95% CI 1.08–1.58, *P* = 0.0056), age > 60 years (HR 1.29, 95% CI 1.08–1.54, *P* = 0.0047), smoking (HR 1.55, 95% CI 1.30–1.86, *P* < 0.0001), ECOG-PS of 2–3 (HR 1.50, 95% CI 1.13–1.99, *P* = 0.0050), squamous carcinoma (HR 1.36, 95% CI 1.10–1.68, *P* = 0.0041), presence of > 2 metastatic sites (HR 1.26, 95% CI 1.01–1.58, *P* = 0.0429), and unknown first-line treatment status(HR 1.52, 95% CI 1.24–1.87, *P* < 0.0001). Notably, both low (HR 1.46, 95% CI 1.10–1.93, *P* = 0.0094) and high FPG levels (HR 1.43, 95% CI 1.11–1.84, *P* = 0.0052) were significantly associated with poor OS. Conversely, EGFR/ALK mutations (HR 0.39, 95% CI 0.28–0.56, *P* < 0.0001) and combination therapy for first-line treatment (HR 0.60, 95% CI 0.38–0.95, *P* = 0.0282) were associated with a reduced risk of mortality. Kaplan-Meier analysis (**Figures 1A, B**) corroborated these findings, demonstrating a significantly shorter median OS in both the low (12.0 *vs*. 18.5 months, *P* = 0.0093) and high FPG groups (14.4 *vs*. 18.5 months, *P* = 0.0049) compared to the normal FPG group.

**Table 2:** Univariate and multivariable Cox regression analysis of factors associated with OS (n=960).

Variables	Univariate analyses		Multivariate analyses	
	HR (95% CI)	P-value	HR (95% CI)	P-value
Male	1.31 (1.08, 1.58)	**0.0056**	0.98 (0.75, 1.26)	0.8553
Age >60 years	1.29 (1.08, 1.54)	**0.0047**	1.11 (0.92, 1.34)	0.2642
Smoking history	1.55 (1.30, 1.86)	**<0.0001**	1.37 (1.07, 1.76)	**0.0138**
ECOG-PS 2-3	1.50 (1.13, 1.99)	**0.0050**	1.18 (0.87, 1.59)	0.2890
BMI (kg/m²)				
≥ 25	Reference			
18.5-24.9	1.16 (0.89, 1.53)	0.2748		
< 18.5	1.19 (0.85, 1.67)	0.3167		
Histological type				
Adenocarcinomas	Reference		Reference	
Squamous carcinoma	1.36 (1.10, 1.68)	**0.0041**	1.26 (1.00, 1.59)	0.0512
Others	1.13 (0.70, 1.81)	0.6159		
Stage IV	1.30 (1.00, 1.69)	0.0532		
Number of metastatic sites				
≤2	Reference		Reference	
>2	1.26 (1.01, 1.58)	**0.0429**	1.41 (1.11, 1.79)	**0.0049**
Unknown	0.89 (0.63, 1.27)	0.5182		
EGFR/ALK mutation				
No	Reference		Reference	
Yes	0.39 (0.28, 0.56)	**<0.0001**	0.46 (0.31, 0.66)	**<0.0001**
Unknown	0.85 (0.68, 1.05)	0.1253		
First-line treatment				
Chemotherapy	Reference		Reference	
TKIs	0.85 (0.66, 1.09)	0.2005		
Combination therapy	0.60 (0.38, 0.95)	**0.0282**	0.57 (0.36, 0.91)	**0.0178**
Unknown	1.52 (1.24, 1.87)	**<0.0001**	1.39 (1.12, 1.73)	**0.0032**
FPG				
Normal	Reference		Reference	
Low	1.46 (1.10, 1.93)	**0.0094**	1.41 (1.06, 1.88)	**0.0196**
High	1.43 (1.11, 1.84)	**0.0052**	1.43 (1.11, 1.85)	**0.0059**

Independent variables with P<0.05 in the univariate analyses were included in the adjusted Cox proportional hazards model; HR, hazard ratio; CI, confidence interval; ECOG PS, Eastern Cooperative Oncology Group Performance Status; BMI, body mass index; EGFR, epidermal growth factor receptor; ALK, Anaplastic Lymphoma Kinase; TKIs, tyrosine kinase inhibitors; FPG, Fasting Plasma Glucose. Combination therapy in this study was defined as chemotherapy combined with anti-angiogenic therapy or immunotherapy. *P* values <0.05 were highlighted in bold.

In the multivariate analysis, after adjusting for all significant univariate predictors (**Table 2**), smoking (HR 1.37, 95% CI 1.07–1.76; *P* = 0.0138), > 2 metastatic sites (HR 1.41, 95% CI 1.11–1.79; *P* = 0.0049), unknown first-line treatment status (HR 1.39, 95% CI 1.12–1.73; *P* = 0.0032), low FPG (HR 1.41, 95% CI 1.06–1.88; *P* = 0.0196), and high FPG (HR 1.43, 95% CI 1.11–1.85; *P* = 0.0059) remained independent predictors of poorer survival. EGFR/ALK mutations (HR 0.46, 95% CI 0.31–0.66; *P* < 0.0001) and combination therapy (HR 0.57, 95% CI 0.36–0.91; *P* = 0.0178) maintained protective effects. These findings indicate that abnormal FPG levels, regardless of whether they are low or high, consistently point to unfavorable survival outcomes in advanced NSCLC patients.

### Smooth curve fitting of FPG in predicting mortality risk

**Figure 2:**
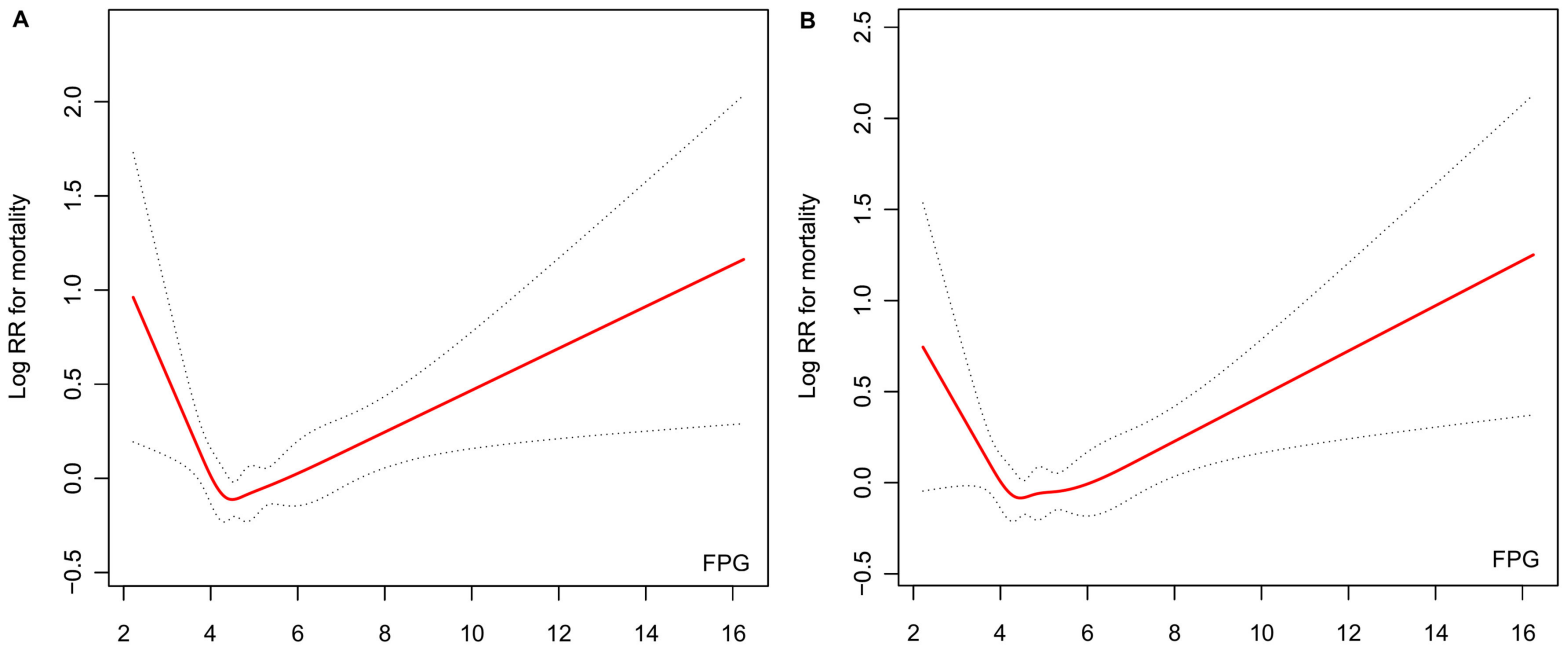
Association between baseline FPG levels and mortality risk. **(A)** Unadjusted smooth curve fitting. **(B)** Smooth curve fitting adjusted for sex, age, smoking status, ECOG-PS, histological classification, number of metastatic sites, EGFR/ALK mutation status, and first-line treatment.

Smoothing curve analysis demonstrated a non-linear association between pretreated FPG and mortality risk. The unadjusted smoothing curve revealed a distinct “V”-shaped relationship (**Figure 2A**), wherein the mortality risk initially decreased with increasing FPG levels but subsequently increased beyond a specific inflection point. This pattern persisted even after adjustment for potential confounders, with the adjusted smoothing curve (**Figure 2B**) mirroring a similar “V”-shaped trend, suggesting the robustness of the observed relationship.

To quantify this nonlinearity, piecewise Cox proportional hazards regression after adjustment was performed, identifying an inflection point at 4.46 mmol/L (**Table 3**). Below this threshold, each 1 mmol/L increase in pretreatment FPG was associated with a 30% reduction in mortality risk. Conversely, above 4.46 mmol/L, each 1 mmol/L rise in FPG corresponded to an 11% increase in mortality risk. The difference in HRs across the inflection point was statistically significant (*P* = 0.0048), confirming distinct risk patterns on either side of the threshold value. Model comparison further validated the superiority of the piecewise Cox model over the linear Cox model (*P* = 0.0070 for the likelihood ratio test), indicating that the nonlinear, threshold-dependent relationship better captured the interplay between pretreatment FPG and mortality risk in this cohort.

**Table 3:** Analysis of threshold effect of FPG (per unit increase) on death risk.

Outcome:	HR (95%CI) *P* value/ *P* value
Model I	1.07 (1.00, 1.13) **0.0481**
Model II	
inflection point (K = 4.46)	
< K	0.70 (0.52, 0.94) **0.0185**
> K	1.11 (1.04, 1.19) **0.0015**
Wald test	**0.0048**
Log likelihood ratio test	**0.0070**

HR (95% CI) *P* value/ *P* value. Model I: Cox proportional hazard model; Model II: two-piecewise Cox proportional hazard model; both models adjusted for sex, age, smoking status, ECOG-PS, histological classification, number of metastatic sites and first-line treatment. The Wald test was used to assess whether there was a significant difference in the HRs on either side of the inflection point (K); Log likelihood ratio test was performed to determine any differences between model I and model II. *P* values less than 0.05 are written in bold.

### Stratified analyses by potential confounders

**Figure 3:**
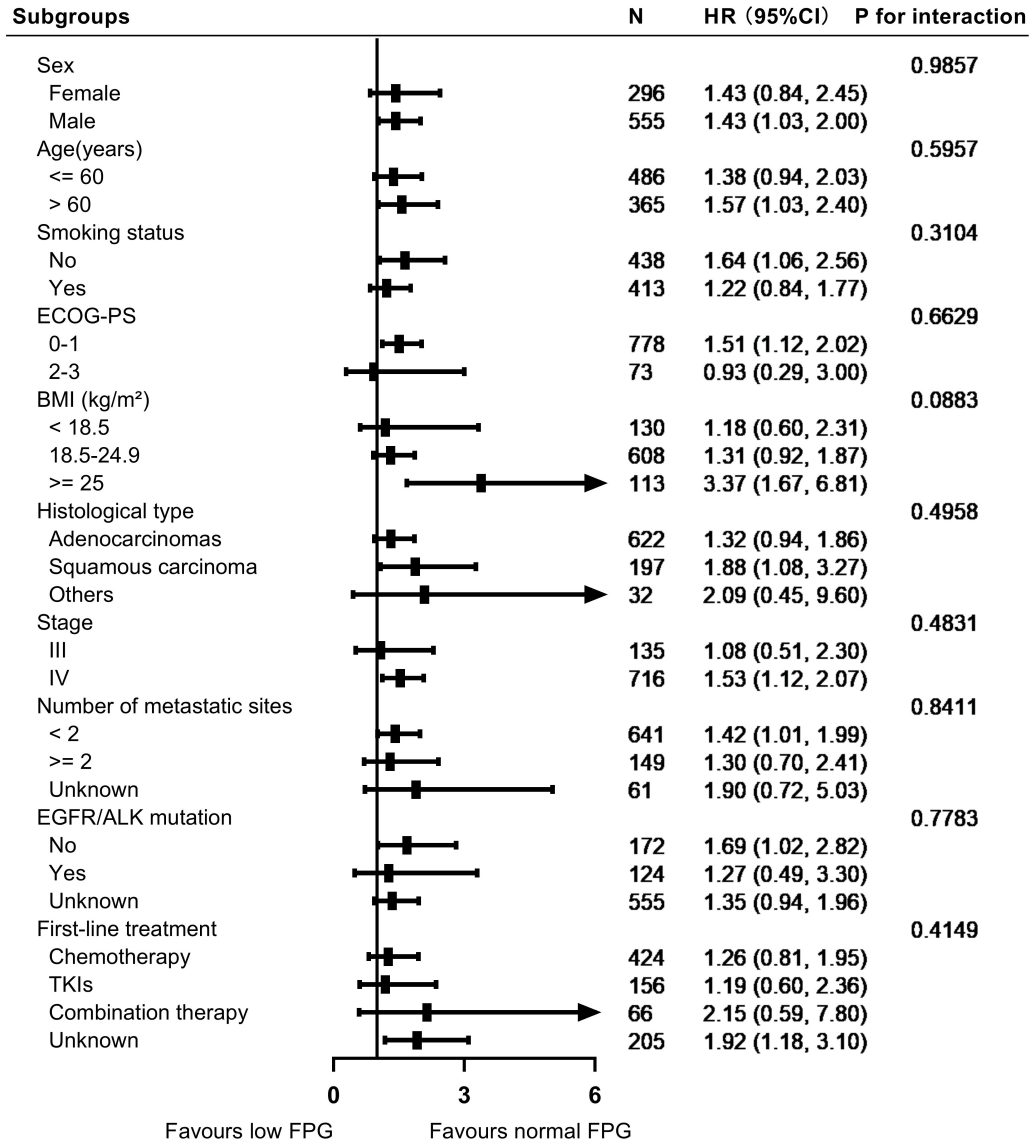
Forest plot showing the impact of low FPG levels on survival outcomes in patients with advanced NSCLC.

**Figure 4:**
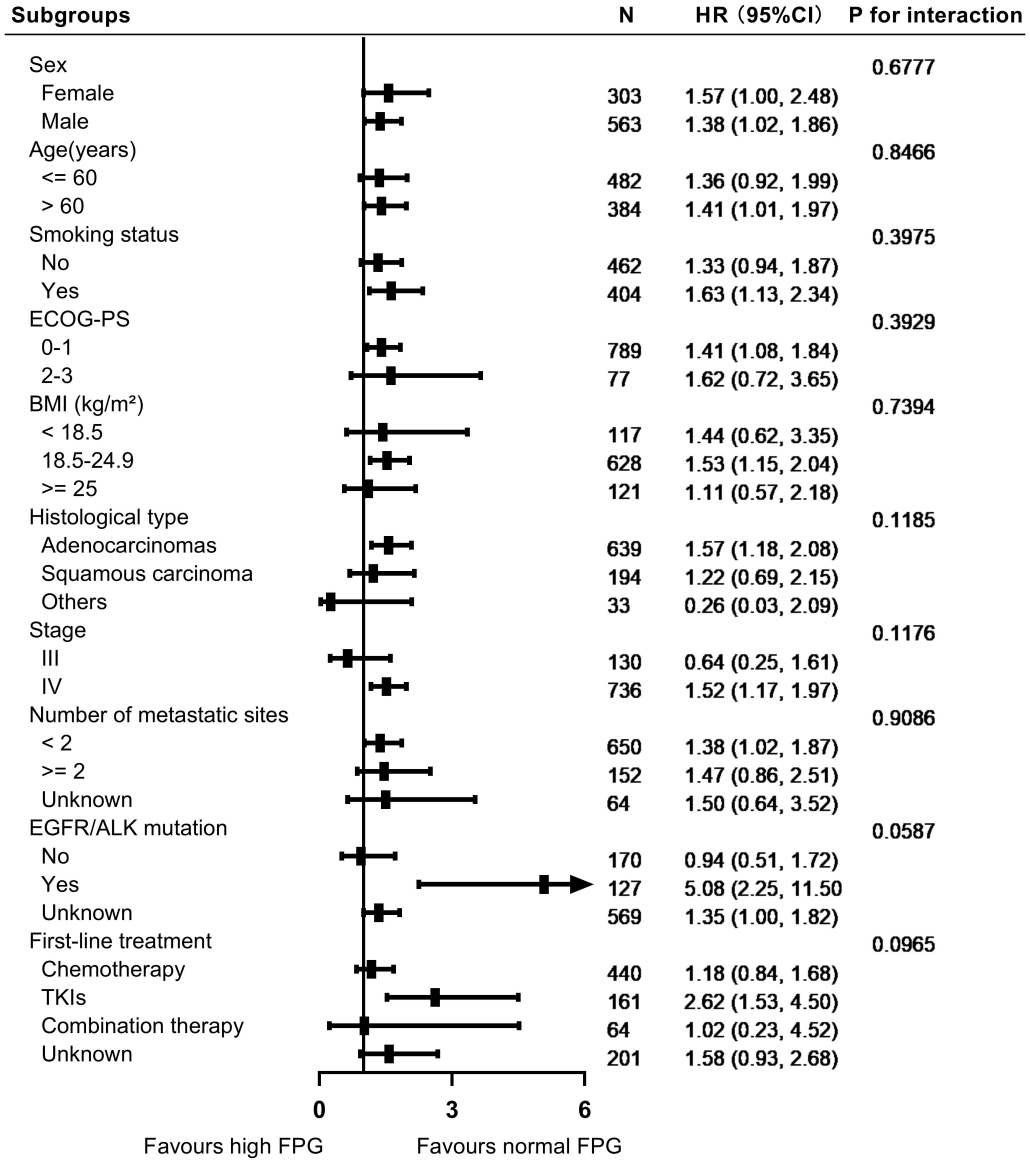
Forest plot showing the impact of high FPG levels on survival outcomes in patients with advanced NSCLC.

In the stratified analyses, we evaluated the impact of FPG levels on overall survival across various subgroups, including sex, age, smoking status, ECOG-PS, histological classification, clinical stage, number of metastatic sites, EGFR/ALK mutation status, and first-line treatment. **Figures 3**, **4** illustrate the subgroup analyses for low and high FPG levels, respectively. Across all subgroups, abnormal FPG levels were associated with a detrimental effect on overall survival. Importantly, the interaction tests did not reveal any significant interactions, indicating that the adverse impact of abnormal FPG levels on survival was consistent across these potential confounders. These results suggest that the adverse prognostic impact of abnormal FPG levels remains robust, irrespective of patient characteristics or treatment factors.

## Discussion

In the baseline characteristics, we observed that older age and elevated BMI were both significantly associated with high FPG levels. This association may reflect the natural progression of metabolic changes with aging. Older individuals with high BMI are more prone to impaired glucose regulation or diabetes due to factors such as insulin resistance, declined beta-cell function, changes in body composition, or decreased physical activity levels that occur with age (18, 19). These factors can elevate FPG levels in older adults and those with a high BMI. In addition, a significant association between low FPG levels and smoking was observed. Smoking has been shown in several studies to influence glucose metabolism. Smokers may have lower FPG levels due to increased insulin sensitivity or changes in energy metabolism caused by nicotine’s pharmacological effects (20, 21). Additionally, smoking-related lifestyle factors, such as reduced appetite or altered eating habits, could also contribute (22). Due to the uneven distribution of age and smoking status among the three FPG groups, adjusting for these confounding factors in the multivariate analysis was essential. This adjustment ensured an accurate assessment of the independent effect of FPG on the long-term prognosis.

FPG is a vital metabolic marker, and its role in influencing outcomes in various malignancies (e.g., breast, endometrial, liver, pancreas, colorectal, and bladder cancers) has received increasing attention (23, 24). In this study, we explored the association between FPG and OS in patients with advanced NSCLC. The baseline FPG levels of all participants were measured using venous blood samples collected uniformly at 5 a.m., minimizing circadian variation and preanalytical bias. Additionally, individuals with conditions that could cause short-term glucose fluctuations, such as acute stress, infection, or recent use of steroid hormones, were excluded, ensuring that the measured FPG values more accurately reflected the long-term glycemic status. By categorizing patients into three groups based on their FPG levels—low (< 3.9 mmol/L), normal (3.9–6.1 mmol/L), and high (> 6.1 mmol/L)—we suggested that both hypoglycemia and hyperglycemia are associated with poorer survival outcomes. Correspondingly, a “V”-shaped relationship between FPG and mortality risk was identified using smooth curve fitting, with FPG treated as a continuous variable. These findings remained consistent across multiple subgroup analyses and statistical models. Our findings contribute to the understanding of the biological and clinical significance of glycemic control in cancer progression and patient management.

The observed relationship between FPG and OS in advanced NSCLC may be explained by several potential mechanistic hypotheses. Repeated or persistent hypoglycemia imposes metabolic stress that selects for tumor cell clones less dependent on glucose and more adapted to fatty acid and glutamine oxidation, thereby enhancing tumor adaptability and invasiveness (25). Low-glucose, low-proliferation signaling can induce a reversible dormant state, reducing sensitivity to cytotoxic agents that primarily target dividing cells and increasing the risk of recurrence (26). Tumor cells, including those in lung cancer, can secrete insulin-like growth factors (IGFs). Binding of IGF to insulin-like growth factor receptor (IGF1R) activates IRS–PI3K–AKT–mTORC1/2 and RAS–RAF–MEK–ERK cascades, promoting cell-cycle progression; protein, lipid, and nucleoside synthesis; inhibition of apoptosis; increased glucose uptake; and metabolic plasticity, collectively driving tumor growth and drug resistance (27). Recurrent hypoglycemia can trigger fluctuations in counter-regulatory hormones (adrenaline, cortisol, glucagon) and compensatory hyperinsulinemia. Insulin and IGF-1 further promote proliferation, survival, and metabolic plasticity through the PI3K–AKT–mTOR axis. Clinically, glycemic variability, hypoglycemia followed by rebound hyperglycemia, induced by exogenous insulin or insulin secretagogues may amplify the tumor-promoting potential of these pathways, in a tumor type–dependent manner (28, 29).

Conversely, hyperglycemia has been linked to enhanced tumorigenesis and worse cancer outcomes through several pathways, including its role as an energy source for rapidly proliferating tumor cells, supporting the Warburg effect, and activating pro-survival pathways such as Akt and mTOR signaling (8, 30, 31). Additionally, hyperglycemia is associated with increased reactive oxygen species production, systemic inflammation, and immune suppression, all of which can adversely affect NSCLC prognosis (11, 32). Beyond the tumor microenvironment, dysregulated glucose metabolism exerts systemic effects. For instance, hyperglycemia often coexists with metabolic comorbidities, such as diabetes or obesity, which are established risk factors for poor outcomes in patients with cancer (10). The interaction between metabolic dysfunction, chronic inflammation, and oxidative stress fosters a pro-tumorigenic environment that accelerates disease progression (11). The V-shaped relationship observed in our study likely underscores the dual burden of advanced disease and the harmful effects of metabolic dysregulation, suggesting that maintaining optimal glucose levels may contribute to improved patient outcomes.

Our findings are consistent with previous reports suggesting a link between glycemic dysregulation and cancer survival. Studies on other malignancies, such as pancreatic, bladder, colorectal, breast, and cervical cancers, have similarly reported poorer survival outcomes associated with high FPG levels (3–7). However, these studies generally did not explore the impact of low FPG levels on prognosis. Currently, the literature specific to NSCLC remains limited. Zhu, L. et al. demonstrated that diabetes was significantly associated with worse overall survival in lung cancer patients, but the analysis did not include patients with impaired fasting glucose. This omission may have led to an incomplete evaluation of the potential impact of blood glucose levels in non-diabetic individuals (33). Bergamino M et al. demonstrated that elevated baseline FPG levels significantly impact overall survival in patients with locally advanced NSCLC undergoing concurrent chemoradiotherapy (34). However, their study did not assess the association between low FPG levels and the clinical outcomes. Currently, research exploring the relationship between different levels of glucose metabolic abnormalities and overall survival in patients with lung cancer remains limited. Compared with previous studies, our study is the first to identify a “V”-shaped relationship between baseline FPG and overall survival. This finding underscores the need for further investigation into the full spectrum of blood glucose levels and their potentially complex associations with cancer prognosis. Such comprehensive research could offer deeper insights into the role of glucose metabolism abnormalities in lung cancer outcomes.

In addition to FPG, other biomarkers of glucose metabolism, such as glycated hemoglobin (HbA1c), insulin, C-peptide, and homeostatic model assessment of insulin resistance (HOMA-IR), have been reported to hold significant prognostic value in NSCLC and could provide complementary perspectives on metabolic status (28, 35, 36). Compared with these markers, FPG is inexpensive, widely available, and routinely measured, making it well-suited for large-scale clinical applications. Importantly, our study highlights a “V”-shaped relationship between FPG and survival in advanced NSCLC, revealing that both hypoglycemia and hyperglycemia are linked to poorer outcomes. This contrasts with other glucose metabolism biomarkers, which predominantly focus on hyperglycemia and do not capture the prognostic implications of low serum glucose levels. Thus, while FPG has limitations in reflecting long-term glycemic trends, its ability to detect both extremes of glycemia allows it to address survival risks associated with hypoglycemia, a gap not filled by other biomarkers.

The findings of this study have several clinical implications. First, FPG, a routinely measured and easily accessible biomarker, could serve as a valuable tool for risk stratification in patients with advanced NSCLC. Identifying patients with extreme FPG levels may help clinicians identify high-risk individuals and tailor interventions. Second, maintaining normoglycemia may represent a novel therapeutic target for improving NSCLC outcomes. While glycemic control is already a cornerstone of diabetes management, our findings suggest that even non-diabetic NSCLC patients may benefit from interventions aimed at optimizing glucose metabolism. For hyperglycemia, strategies such as lifestyle modifications, dietary interventions, and anti-diabetic medications can be explored. For hypoglycemia, ensuring adequate nutritional support and addressing the underlying causes, such as systemic inflammation or cachexia, may be critical. Finally, our results underscore the need for integrated care models in oncology that address tumor biology, metabolic health, and nutritional health. The intersection of metabolic health and cancer outcomes represents an emerging field that warrants further investigation to refine the prognostic tools and identify novel therapeutic opportunities.

This retrospective cohort study had several limitations. First, the potential for selection bias and residual confounding factors cannot be entirely ruled out, despite the adjustments made in the analysis. Second, the single-center design inherently limits the generalizability of the findings to other populations or healthcare settings, and external validation cohorts will be necessary in future research. Third, the sample size, particularly within certain subgroups, may have limited the statistical power to detect smaller but potentially meaningful differences in survival outcomes. Finally, While these associations persisted after adjustment for multiple covariates, the observational nature of the study, single-center design, and measurement of FPG at only one time point limit the ability to infer causality.

## Conclusion

This real-world study demonstrated that abnormal FPG levels, both low (< 3.9 mmol/L) and high (> 6.1 mmol/L), serve as independent prognostic risk factors for reduced overall survival in patients with advanced NSCLC. A non-linear, “V”-shaped relationship existed between pre-treatment FPG and mortality risk, with the lowest risk observed within the normal FPG range (3.9–6.1 mmol/L). These findings suggest that FPG may have potential as a prognostic marker in advanced NSCLC, but further prospective, multi-center studies incorporating additional metabolic biomarkers are warranted to validate these results and clarify underlying mechanisms before FPG can be integrated into routine prognostic assessment or targeted interventions.

## Data Availability

The raw data supporting the conclusions of this article will be made available by the authors, without undue reservation.

## Supplementary material

Supplementary Figure 1 Flow chart for screening eligible patients.
