# Supplementary figures and images for: Impact of pre-therapeutic fasting plasma glucose on survival outcomes in advanced non-small cell lung cancer patients
Source: Front Endocrinol (Lausanne). 2026 Jan 9;16:1630503. doi: 10.3389/fendo.2025.1630503 (PMC12827117; doi:10.3389/fendo.2025.1630503)

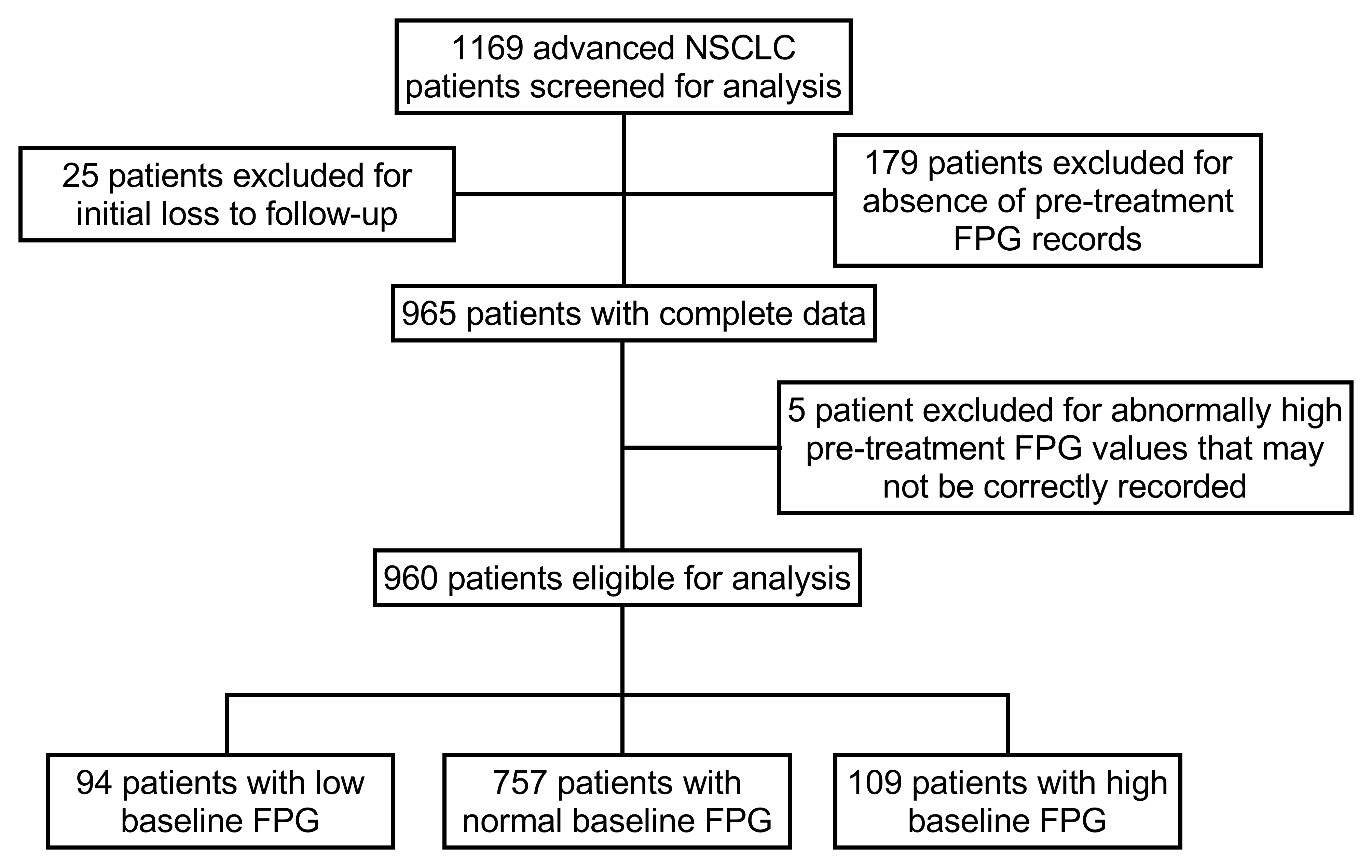

Supplement: Supplementary Figure 1 — Flow chart for screening eligible patients. [file Image1.tif]
